# Social relationships enhance the time spent eating and intake of a novel diet in pregnant Hanwoo (*Bos taurus coreanae*) heifers

**DOI:** 10.7717/peerj.3329

**Published:** 2017-05-09

**Authors:** Dong-Han Shin, Hyun-Min Kang, Seongwon Seo

**Affiliations:** Division of Animal and Dairy Science, Chungnam National University, Daejeon, Republic of Korea

**Keywords:** Eating behavior, Feed intake, Hanwoo (*Bos Taurus coreanae*), Social relationships, Pregnant heifer

## Abstract

The objective of this study was to evaluate the effects of social relationships on the feed intake, eating behavior, and growth, upon exposure to a novel diet, in Hanwoo (*Bos taurus coreanae*) heifers during pregnancy. Twenty-four pregnant Hanwoo heifers, averaging 438 ± 27.8 kg in weight, 21 months in age, and 194 ± 8.5 days in pregnancy, were involved in a two-month (eight weeks) experiment. The heifers were randomly assigned to either the single housing group (SG; one individual per pen, *n* = 12), or the paired housing group (PG; two individuals per pen, *n* = 12). All pens were of the same size (5 × 5 m) and provided with one feed bin, which automatically recorded the individual feed intake and eating behavior. As the experiment began, the diet of the heifers was switched from a total mixed ration (TMR; 250 g/kg ryegrass straw and 750 g/kg concentrate mix) to a forage-only diet (mixed hay cubes composed of 500 g/kg alfalfa, 250 g/kg timothy, and 250 g/kg blue grass hay). The heifers were fed ad libitum twice a day. The individual feed intake and eating behavior were recorded daily throughout the experiment, and body weights (BWs) were measured every four weeks before the morning feeding. PG animals visited the feed bin 22% less often than SG. PG, however, stayed 39% longer in the feed bin and consumed 40% more feed per visit, compared with SG. Consequently, PG heifers spent 23% more time in eating and had 16% more daily dry matter intake than SG during the experiment. Average daily gain during the experimental period tended to be greater in PG than in SG. When pregnant Hanwoo heifers encountered a novel diet, social relationships (i.e., presence of a pen-mate) enhanced their time spent eating and feed intake. Social interactions, even with an unfamiliar individual, may be helpful for pregnant Hanwoo heifers cope with a diet challenge compared to solitary situation.

## Introduction

Cattle are social animals that live in groups under natural or semi-natural conditions. From an evolutionary perspective, group living aids in detecting, acquiring, and defending food, as well as avoiding predators. On the other hand, it has disadvantages such as competition, conspicuousness toward predators, and susceptibility toward infection (Mendl & Held, 2001). Within a group, cattle communicate with each other agonistically (e.g., aggression and competition) to establish social dominance, and non-agonistically (e.g., allogrooming and licking) to reduce tension, reinforce alliances, and stabilize social relationships (Bouissou et al., 2001). The intensive farming system often ignores the effects of social relationships among cattle, and the methods for group housing are mainly determined by animal productivity, ease of management, and farm economics (Val-Laillet et al., 2009). An individual animal might get disturbed by a farm procedure, such as re-grouping, diet change, or moving, which may result in discomfort and poor productivity (Bøe & Færevik, 2003; Grant & Albright, 2001). During such disruptive farm practices, the social interactions can be a source of stress, but can also help an individual cope with other stresses (Broom, 1991). For example, compared with individually housed calves, group-housed calves appeared to be less afraid in a new environment (Jensen et al., 1997; Veissier et al., 1994). Thus, understanding the impact of social interactions on the welfare and productivity of cattle is crucial for implementation of better farm practices.

Several studies have investigated the effect of group vs. individual housing in cattle, most of which have focused on calves. Earlier studies have suggested that individual feeding is superior to group feeding in veal calves in terms of growth, behavior, health, and disease control (Maatje et al., 1993; Van Putten, 1982). Chua et al. (2002), however, reported that pair-housed calves remained healthier and gained weight faster than the individually housed ones before and after weaning. Other recent studies have also shown a positive response to group housing in terms of feed intake and/or weight gain of calves (Bernal-Rigoli et al., 2012; Costa et al., 2015; De Paula Vieira, Von Keyserlingk & Weary, 2010; Miller-Cushon & DeVries, 2016), although negative responses have also been reported (Terré, Bach & Devant, 2006). However, there are only a few studies on adult cattle, most of which involve dairy cattle. Negative effects of grouping (e.g., competition for feed and aggressive interactions for social ranking) have been reported, which usually intensify with increasing stocking density and decreasing the number or area of feed bins per animal (Friend, Polan & McGilliard, 1977; González et al., 2008). In these situations, dairy heifers alter their feeding behavior (DeVries & von Keyserlingk, 2009). For example, first-calf dairy heifers, when reared separately from older cows, increased their dry matter intake (DMI), eating time, and meal frequency (Konggaard & Krohn, 1978; cited in Grant & Albright, 2001). However, little is known about the response of beef cattle during pregnancy. During pregnancy, cattle may exhibit abnormal feeding behavior and are particularly susceptible to metabolic disorders, especially when they experience sudden social and environmental changes (Grant & Albright, 1995). Hanwoo is an indigenous Korean breed, known for yielding high quality marbled beef. Group housing is common in Hanwoo heifers except during the late gestation period when they are individually housed. In the aspect of animal welfare, the housing system of Hanwoo pregnant heifers had become of interest, and a better understanding of their social behavior is essential.

Therefore, the objective of this study was to evaluate the effects of social relationships on the feed intake, eating behavior, and growth of Hanwoo (*Bos Taurus coreanae*) pregnant heifers in a new diet regimen. Twenty-four pregnant Hanwoo heifers, randomly assigned to either single or pair housing, were tested for feeding behavior, feed intake, and growth, using a novel diet. The results from this study suggest that the social interactions may be helpful for pregnant Hanwoo heifers to cope with the challenges posed by a novel diet.

## Materials and Methods

This study was conducted at Center for Animal Science Research, Chungnam National University, Republic of Korea. The use of animals and the protocols for this experiment were reviewed and pre-approved by the Chungnam National University (CNU) Animal Research Ethics Committee (CNU-00649).

### Experimental design, animals, and diets

In order to evaluate the effects of social interactions in pregnant native Korean heifers, an eight-week experiment was performed. This study focused on the differences in DMI, eating behavior (i.e., frequency—the number of visits to the feed bin per day, DMI per meal, time spent eating per visit and per day (min), eating duration per visit (min), and eating rate (g/min)), and growth performance (i.e., average daily gain (ADG) and feed conversion ratio (FCR)) caused by the housing condition (single vs. paired). The results might be insufficient to understand the complete physiological response to social interactions. Nonetheless, any differences in feeding behavior and growth should be particularly interesting for field application.

Twenty-four pregnant heifers participated in this study. The animals averaged 438 ± 27.8 kg in BW, were 21 months old, and were 194 ± 8.5 days pregnant on an average. Before the onset of the experiment, the heifers had been housed in pairs with other heifer that were not participants in this study and fed a TMR composed of 250 g/kg ryegrass straw and 750 g/kg concentrate mix. Individual heifers were then randomly assigned to one of the following treatments: (a) SG (one heifer per pen, *n* = 12); and (b) PG (two heifers per pen, *n* = 12). The PG heifers were paired with a novel partner.

Each pen was of the same size (5 × 5 m) and had one feed bin. Because the opening of the feed bin allows only one animal, one heifer at a time can gain access to each feed bin. Each feed bin was equipped with a real-time electronic individual feeding system that recognized each heifer entering the feeder by sensing the radio-frequency identification (RFID) tag attached to each animal (Dawoon Co., Incheon, Republic of Korea). The individual feeding system determined the amount of feed consumed per visit by measuring the difference between the weights of the feeder before and after a visit. Daily feed intake was the sum of the amounts of feed consumed per visit during 24 h. Eating behavior of each heifer (i.e., the number of visits to the feed bin per day and the time spent at the feed bin per visit) was also automatically recorded by the system.

The heifers were fed with the same forage-only diet, mixed hay cubes (873 g/kg DM) composed of 500 g/kg alfalfa, 250 g/kg timothy, and 250 g/kg bluegrass hay. The analyzed chemical composition of the TMR and the mixed hay cubes are described in Table 1. The diet was given ad libitum two times a day at 08:00 and 18:00 h. The heifers had unrestricted access to drinking water during the experiment.

**Table 1:** Analyzed chemical composition (g/kg DM unless otherwise stated) of the experimental diets.

Item	Total mixed ration^1^	Mixed hay cubes^2^
DM, g/kg of as fed	892	873
OM	911	840
CP	139	141
EE	42	17
aNDF	378	524
ADL	56	81
NEm, MJ/kg of DM	7.3	4.0
NEg, MJ/kg of DM	4.8	1.8

**Notes:**

DM, dry matter; OM, organic matter; CP, crude protein; EE, ether extract; aNDF, neutral detergent fiber analyzed using a heat stable amylase and expressed inclusive of residual ash; ADL, acid detergent lignin; NEm, net energy for maintenance; NEg, net energy for growth.

1 250 g/kg ryegrass straw and 750 g/kg concentrate mix.

2 500 g/kg alfalfa, 250 g/kg timothy, and 250 g/kg bluegrass hay.

### Measurement and chemical analyses

The BW of the heifers was measured every four weeks before the morning feeding, thus, minimizing any adverse effect caused by BW measurement.

The diet (i.e., mixed hay cubes) was sampled several times during the experimental period. The samples were dried at 60 °C for 96 h, ground through a cyclone mill (Foss, Hillerød, Denmark) fitted with a 1 mm screen, and pooled for chemical analyses. Contents of dry matter (DM; #934.01), crude protein (CP; #976.05), ether extract (#920.39), acid detergent fiber (#973.18), and ash (#942.05) were determined as per the protocols described by AOAC International (2005). CP was calculated as 6.25 times the nitrogen content, and the total nitrogen was estimated by the Kjeldahl method using a DK 20 Heating Digester and Semi-Automatic Distillation Unit, Model UDK 139 (VELP Scientifica, Usmate, Italy). Acid detergent lignin and neutral detergent fiber were analyzed as described by Van Soest, Robertson & Lewis (1991). Neutral detergent insoluble CP and acid detergent insoluble CP were also determined as described by Licitra, Hernandez & Van Soest (1996). The energy values (i.e., TDN, NEm, and NEg) were calculated using National Research Council (2001) equations.

### Statistical analysis

The experimental unit of this study was an individual heifer. One of the SG heifers had a health problem during week 4, and was removed from the study. As a result, the total numbers of experimental units for statistical analysis were 12 and 11 for PG and SG, respectively.

In order to test the possibility that differences in the feeding behavior of heifers between two treatments might be reduced by time due to adaptation, the monthly (i.e., two 4-week periods) data of heifers’ growth performance and eating behavior were analyzed using the PROC GLM procedure of SAS 9.2 (SAS Institute Inc., Cary, NC, USA). The linear model was as follows:

$$y_{ijk} = \mu + \tau_{i} + \rho_{j} + \tau\rho_{ij}+e_{ijk}$$

where, *y_ijk_* is *k*th observation for each response variable (*k* = 1–12) in *i*th treatment (*i* = 1 and 2) and *j*th month (*j* = 1 and 2), μ is the overall mean, τ_*i*_ is the fixed effect of *i*th treatment, ρ_*j*_ is the fixed effect of *j*th month, is the fixed effect of interaction between treatment and month, and *e_ijk_* is the unexplained random effect on *k*th observation in *i*th treatment and *j*th month.

Significance was declared at *P* < 0.05 and tendencies were discussed at 0.05 ≤ *P* < 0.10.

## Results

### Feed intake and eating behavior

Dry matter intake significantly differed by both housing and period. DMI was greater in PG, compared with SG (Table 2). Average DMI for the first month was significantly higher in PG compared with SG (7.2 vs. 6.1 kg/day, respectively; *P* < 0.05). DMI of PG (8.4 kg/day) was greater than that of SG (7.3 kg/day) during the second month as well (*P* < 0.05). Consequently, the overall daily DMI was significantly higher by 1.1 kg/day in PG compared with SG (7.8 vs. 6.7 kg/day, respectively; *P* < 0.001).

**Table 2:** Effect of single vs. paired housing on feed intake and eating behavior of pregnant Hanwoo heifers.

Item	First month		Second month		SEM^3^	*P* value		
	PG^1^	SG^2^	PG	SG		Housing	Month	Interaction
DMI, kg	7.2^b^	6.1^c^	8.4^a^	7.3^b^	0.28	<0.001	<0.001	0.928
Eating frequency (meals/day)	26.3	31.0	23.3	32.8	3.09	0.026	0.844	0.448
DMI per meal (g/meal)	294^ab^	223^b^	408^a^	280^ab^	42.5	0.024	0.051	0.511
Time spent eating (min/day)	170^ab^	126^b^	191^a^	167^ab^	12.5	0.015	0.027	0.478
Eating duration (min/meal/day)	6.8^ab^	4.7^b^	8.9^a^	6.6^ab^	0.93	0.025	0.037	0.872
Eating rate (g/min)	43.9	60.9	45.2	47.4	5.93	0.114	0.315	0.221

**Notes:**

DMI, dry matter intake.

1 Paired housing group (*n* = 12).

2 Single housing group (*n* = 11).

3 Standard error of the mean.

a–c Mean that do not have common superscripts differ (*P* < 0.05).

Eating behavior of the pregnant heifers for a novel diet significantly differed between PG and SG (Table 2). PG animals visited the feed bin less often than the ones in SG during the experimental period (24.8 vs. 31.9 meals/day; *P* = 0.026). During each visit, however, PG individuals stayed longer in the feed bin compared with the SG animals (7.8 vs. 5.7 min/meal; *P* = 0.025). As a result, PG animals spent significantly more time in eating than the ones in SG (181 vs. 147 min/day; *P* = 0.015) and consumed 100 g more of feed DM per meal (351 vs. 251 g/meal; *P* = 0.024) on average. There was no significant difference in eating rate (g/min) between PG and SG during the experimental period (*P* > 0.10).

Compared with the first month, the heifers spent more time in eating (148 vs. 179 min/day; *P* = 0.027) and stayed longer time per a visit to feed bin (5.7 vs. 7.8 min/meal; *P* = 0.037) in the second month. Consequently, DMI per meal tended to increase from 258 g/meal in the first month to 344 g/meal in the second month (*P* = 0.051). There was no difference in eating frequency between the two months.

### Growth performance

The initial and final BW did not differ between PG and SG throughout the experimental period (*P* > 0.10; Table 3). Overall, ADG tended to increase with social grouping, compared with solitary housing (819 vs. 664 g/day for PG and SG, respectively; *P* = 0.083). Feed conversion ratio, an indicator of feed efficiency and calculated as DMI (g) divided by ADG (g), was not significantly different by housing (*P* > 0.10).

**Table 3:** Effect of single vs. paired housing on body weight and feed efficiency of pregnant Hanwoo heifers.

Item	First month		Second month		SEM^3^	*P* value		
	PG^1^	SG^2^	PG	SG		Housing	Month	Interaction
Initial BW, kg	437^b^	438^ab^	471^a^	463^ab^	9.0	0.694	0.002	0.647
Final BW, kg	471	463	484	475	9.5	0.409	0.190	0.979
ADG, g	1,162^a^	876^a^	463^b^	445^b^	85.4	0.083	<0.001	0.124
FCR	6.7^b^	7.3^b^	23.4^a^	13.6^ab^	3.73	0.224	0.004	0.177

**Notes:**

BW, body weight; ADG, average daily gain; FCR, feed conversion ratio calculated as dry matter intake (g) divided by ADG (g).

1 Paired housing group (*n* = 12).

2 Single housing group (*n* = 11).

3 Standard error of the mean.

a,b Mean that do not have common superscripts differ (*P* < 0.05).

Compared to the first month, ADG was decreased and FCR was increased in the second month, mainly due to the shift of diet from high concentrate (250 g/kg ryegrass straw and 750 g/kg concentrate mix) to forage-only.

## Discussion

In the past, animal agriculture was known as animal husbandry. Husbandry means putting the animals into the best possible environment according to their biological needs and nature (Rollin, 2002). As intensive farming practices have become increasingly common, animals’ needs are often ignored in order to increase the productivity. However, animal welfare has become an important public issue, and thus, housing and management practices for livestock animals need to take into consideration their behavior and well-being (Harper & Makatouni, 2002). The first step toward better animal welfare is to understand the animals’ needs (Boissy et al., 2007). Some of these needs are relatively simple, such as nutrition and health related needs. Others are complex and may be as important as nutrition and health for the survival and reproduction of the animals. For example, insufficient contact with other individuals can cause deficiencies in mental functioning (Broom, 1991).

To the best of our knowledge, this is the first study that explores the effects of social grouping compared with solitary housing on the eating behavior, feed intake, and growth of beef heifers during pregnancy. Although feeding behavior and growth measurements alone cannot explain all the underlying physiological responses and mechanisms, they can serve the crucial purpose of directing field application. For a novel diet, the SG individuals visited the feed bin more often than the PG ones. However, these visits were shorter in duration and they consumed less of the novel diet than the PG animals. Consequently, feed intake was greater for PG than SG throughout the experimental period. A higher DMI resulted in a tendency of an increased in ADG in PG. Greater ADG during the first month might be a carry-over effect from consuming the previous concentrate-based TMR.

Pair-housed pregnant Hanwoo heifers seemed to cope with the novel diet stress better than the single-housed heifers. Because the heifers were new to the mixed hay cubes, they might have an initial period of avoidance of the novel diet. Ruminants typically treat anything new with caution and are therefore, reluctant to consume novel foods and explore unfamiliar situations (Burritt & Provenza, 1997; Chapple & Lynch, 1986; Villalba, Provenza & Manteca, 2010). They consume only a small amount of a feed if it is unfamiliar (Launchbaugh, Provenza & Werkmeister, 1997). However, the avoidance of a novel diet can be overcome by “social facilitation” (Ralphs & Olsen, 1990). Social facilitation means that an animal can stimulate the expression of a behavioral response of another animal (Curtis & Houpt, 1983; Weiss & Miller, 1971). By social facilitation, cows consume more in groups than when they are solitary (Grant & Albright, 1995, 2001). Phillips (2004) found that grass intake and time spent eating grass were greater for grouped calves than for solitary calves. De Paula Vieira, Von Keyserlingk & Weary (2010) reported that group housed calves consumed more overall DMI than individually housed calves. In addition, Costa et al. (2014) observed that the paired housed calves had a shorter latency to consuming the starter, and made more frequent visits to the feed than the single housed calves. They concluded that the calves raised in complex social environments might be better able to respond to other changes in their environment. Not only toward a novel diet, but also for environmental and social novelty, pair-housed calves were less reactive and more stable than the individually housed calves (De Paula Vieira, de Passillé & Weary, 2012).

Encountering a new partner can be stressful, but it does not take long to establish the social hierarchy in a group of cattle. Some studies showed changes in behavior and production by regrouping diminished within a day or two (Brakel & Leis, 1976; von Keyserlingk, Olenick & Weary, 2008); others indicated re-grouped cattle return to normal state between one and two weeks (Gupta et al., 2008; Hasegawa et al., 1997; Kondo et al., 1984; Phillips & Rind, 2001). Thus, the re-grouping stress itself may have a short-term and minor effect on cattle behavior and production.

Grouping can be stressful to cattle if they need to compete for feed. Competition for the feed may decrease the time spent eating, and increase the meal frequency and eating rate. Friend, Polan & McGilliard (1977) reported a 33% decrease in the time spent eating a TMR when the length of the feed bunk per cow was reduced from 0.5 to 0.1 m. Compared with the cows that had a feed bin to themselves, the cows that had to share a feed bin with three other cows shortened their eating time by 19% and increased their eating rate by 27% (Olofsson, 1999). Calves also altered their feeding behavior in order to cope with the competition for feed. Miller-Cushon et al. (2014) reported a significantly higher rate of intake (g/min) in competitive feeding (one teat and one feed bucket per two calves) than in non-competitive feeding (two teats and two feed buckets per two calves). In pre-pubertal Holstein heifers, the competitively fed heifers (two heifers per feed bin) reduced their meal frequency, increased the meal duration, apart from tending to shorten the time spent eating and increase the meal size, compared with the non-competitively fed heifers (one heifer per feed bin) (DeVries & von Keyserlingk, 2009). An increase in competition by increasing stocking density (from two heifers per pen to eight heifers per pen) decreased the concentrate eating time, and increased the eating rate and susceptibility to metabolic disorder (González et al., 2008). When social stresses were reduced by separating first-calf heifers from older cows, they increased their eating time, meal frequency, and DMI by 11.4%, 8.5%, and 8.8%, respectively (Konggaard & Krohn, 1978; cited in Grant & Albright, 2001). In the present study, the paired heifers did not compete for feed. They seemed to take turns peacefully and let the partner stay in the feed bin long enough to consume sufficient amount of feed. The diet was offered ad libitum, so feed was available to the animals at any time during the day. In addition, the diet was composed of only forage; cattle spend more time in ruminating than in eating as the fiber concentration increases in the diet (Beauchemin & Buchanan-Smith, 1990). In addition, the digestibility of the mixed hay used in our study was relatively high, so that the heifers spent less time for consuming the feed than other diet (Moon, Lee & Lee, 2004).

Social interactions may result in comfort and better productivity of cattle. Isolation may magnify the stress caused by a novel environment and diet. Social relationships are often beneficial in reducing the effects of stress (Bouissou et al., 2001). For example, social interaction could stop a stereotypy in an animal (Broom, 1991). Sato (1984) reported that live weight gain of weaned calves was positively related with the time they spent receiving licking. Social grooming might also reduce tension and thus, have positive effects on the psychological stability of cattle (Sato, Tarumizu & Hatae, 1993).

There are also some possible limitations in our study. Since detailed biochemical or physiological responses were not investigated, underlying mechanisms of the differences are still to be elucidated in future studies. In addition, the heifers were housed in pairs before the onset of the experiment, and thus the effects of single- vs. pair-housing observed in this study was mainly due to those of isolation vs. new partnership. In order for a more complete test for the effect of social relationships, three additional groups might need to be included in a future study: one that is singly housed throughout the experiment, another that is moved from single- to pair-housing, the other that is kept in a pair during the experiment.

In conclusion, when pregnant Hanwoo heifers encounter a novel diet, social relationships (i.e., presence of a pen-mate), compared with isolation, enhanced the time they spent eating and their feed intake and tended to promote the growth of the animals without altering the feed efficiency.

## Supplemental Information

10.7717/peerj.3329/supp-1 Supplemental Information 1 Supplemental Dataset.

## Additional Information and Declarations

### Data Availability

The following information was supplied regarding data availability: The raw data has been supplied as Supplemental Dataset Files.
