# Supplementary material for: Social relationships enhance the time spent eating and intake of a novel diet in pregnant Hanwoo (Bos taurus coreanae) heifers
Source: PeerJ. 2017 May 9;5:e3329. doi: 10.7717/peerj.3329 (PMC5426355; doi:10.7717/peerj.3329)
Supplement: Supplemental Information 1 [file peerj-05-3329-s001.pdf]

Animal	Period	Treat	BW_initial	BW_final	ADG	DMI	FCR	eat_freq
1	1	pair	416.0	447.5	1086.0	6.6	6.0	29.36
5	1	pair	384.5	400.0	534.0	6.2	11.6	32.43
6	1	single	380.0	414.0	1172.0	7.0	6.0	35.32
9	1	single	441.0	472.0	1069.0	7.1	6.6	45.71
11	1	pair	432.5	476.5	1517.0	7.3	4.8	23.89
12	1	single	506.0	525.0	655.0	5.4	8.3	21.36
13	1	pair	455.0	480.0	862.0	7.2	8.4	25.93
14	1	pair	432.0	466.0	1172.0	7.5	6.4	17.18
15	1	pair	432.0	478.0	1586.0	6.4	4.0	20.86
22	1	single	452.0	475.5	810.0	6.6	8.1	43.61
25	1	single	429.0	441.0	414.0	3.4	8.2	17.75
26	1	single	425.0	452.5	948.0	5.5	5.8	39.64
28	1	pair	440.0	483.0	1483.0	7.2	4.9	14.25
30	1	single	458.0	485.0	931.0	6.3	6.7	28.46
36	1	pair	460.0	489.0	1000.0	6.8	6.8	36.29
45	1	pair	435.5	475.5	1379.0	8.8	6.4	23.61
47	1	single	450.5	486.0	1224.0	7.4	6.0	41.86
48	1	pair	464.0	510.5	1603.0	8.1	5.1	30.86
49	1	pair	445.0	472.5	948.0	7.3	7.7	32.46
50	1	single	377.5	393.0	534.0	5.5	10.4	33.96
54	1	single	446.5	476.5	1034.0	6.3	6.1	12.71
63	1	single	448.0	472.5	845.0	6.5	7.6	21.14
64	1	pair	447.5	470.0	776.0	6.7	8.7	28.25
1	2	pair	447.5	455.0	268.0	7.6	28.4	34.12
5	2	pair	400.0	409.5	339.0	6.7	19.8	36.24
6	2	single	414.0	422.0	286.0	7.8	27.3	55.08
9	2	single	472.0	487.0	536.0	8.0	14.9	58.94
11	2	pair	476.5	500.0	839.0	9.0	10.7	19.80
12	2	single	525.0	539.5	518.0	6.6	12.7	19.64
13	2	pair	480.0	490.5	375.0	8.5	22.7	27.68
14	2	pair	466.0	485.5	696.0	8.5	12.2	17.80
15	2	pair	478.0	488.0	357.0	8.5	23.8	15.56
22	2	single	475.5	494.5	679.0	7.7	11.3	36.92
25	2	single	441.0	463.5	804.0	7.4	9.2	28.12
26	2	single	452.5	460.5	286.0	6.2	21.7	38.64
28	2	pair	483.0	486.5	125.0	8.9	71.2	14.36
30	2	single	485.0	490.0	179.0	7.8	43.6	24.56
36	2	pair	489.0	499.0	357.0	7.3	20.4	32.48
45	2	pair	475.5	496.5	750.0	10.1	13.5	20.92

47	2	single	486.0	496.5	375.0	8.5	22.7	36.88
48	2	pair	510.5	520.0	339.0	9.8	28.9	14.52
49	2	pair	472.5	490.0	625.0	8.3	13.3	26.48
50	2	single	393.0	404.5	411.0	5.8	14.1	29.92
54	2	single	476.5	504.0	982.0	8.3	8.5	10.00
63	2	single	472.5	468.0	-161.0	5.8	-36.0	22.16
64	2	pair	470.0	483.5	482.0	7.5	15.6	19.60
1	Overall	pair	416.0	455.0	684.0	7.0	10.3	31.60
5	Overall	pair	384.5	409.5	439.0	6.5	14.7	34.23
6	Overall	single	380.0	422.0	737.0	7.4	10.0	44.64
9	Overall	single	441.0	487.0	807.0	7.6	9.4	50.71
11	Overall	pair	432.5	500.0	1184.0	8.1	6.9	21.96
12	Overall	single	506.0	539.5	588.0	6.0	10.2	20.55
13	Overall	pair	455.0	490.5	623.0	7.8	12.6	26.75
14	Overall	pair	432.0	485.5	939.0	7.9	8.5	17.47
15	Overall	pair	432.0	488.0	982.0	7.4	7.5	18.04
22	Overall	single	452.0	494.5	746.0	7.0	9.4	40.45
25	Overall	single	429.0	463.5	605.0	5.3	8.7	23.04
26	Overall	single	425.0	460.5	623.0	5.8	9.3	39.17
28	Overall	pair	440.0	486.5	816.0	8.0	9.8	14.30
30	Overall	single	458.0	490.0	561.0	7.0	12.5	26.62
36	Overall	pair	460.0	499.0	684.0	7.0	10.3	34.49
45	Overall	pair	435.5	496.5	1070.0	9.4	8.8	22.34
47	Overall	single	450.5	496.5	807.0	7.9	9.8	39.51
48	Overall	pair	464.0	520.0	982.0	9.0	9.1	23.15
49	Overall	pair	445.0	490.0	789.0	7.8	9.8	29.64
50	Overall	single	377.5	404.5	474.0	5.7	12.1	32.06
54	Overall	single	446.5	504.0	1009.0	7.3	7.2	11.43
63	Overall	single	448.0	468.0	351.0	6.2	17.6	21.62
64	Overall	pair	447.5	483.5	632.0	7.1	11.2	24.17

eat_dur	meal_dur	eat_rate	DMImeal
165.15	5.63	39.67	223.18
144.38	4.45	42.82	190.66
111.74	3.16	62.76	198.56
75.89	1.66	93.64	155.43
191.11	8.00	38.15	305.12
155.26	7.27	35.07	254.93
194.34	7.50	37.04	277.60
140.12	8.16	53.35	435.12
143.67	6.89	44.32	305.19
83.22	1.91	78.73	150.25
23.69	1.33	144.11	192.36
150.64	3.80	36.76	139.67
119.53	8.39	60.22	505.11
238.81	8.39	26.28	220.45
183.54	5.06	37.21	188.19
233.50	9.89	37.54	371.35
150.80	3.60	48.95	176.37
125.35	4.06	64.78	263.17
231.41	7.13	31.50	224.56
99.04	2.92	55.90	163.02
121.39	9.55	51.69	493.54
180.93	8.56	35.70	305.52
167.73	5.94	40.16	238.46
190.92	5.60	39.81	222.74
142.68	3.94	46.96	184.88
93.28	1.69	83.62	141.61
225.49	3.83	35.48	135.73
227.73	11.50	39.52	454.55
174.69	8.89	37.78	336.05
215.14	7.77	39.51	307.08
198.79	11.17	42.76	477.53
141.43	9.09	60.10	546.27
96.43	2.61	79.85	208.56
186.64	6.64	39.65	263.16
184.85	4.78	33.54	160.46
156.10	10.87	57.02	619.78
215.77	8.79	36.15	317.59
196.06	6.04	37.23	224.75
255.92	12.23	39.46	482.79

171.74	4.66	49.49	230.48
162.42	11.19	60.34	674.93
246.42	9.31	33.68	313.44
115.48	3.86	50.22	193.85
181.86	18.19	45.64	830.00
190.15	8.58	30.50	261.73
161.12	8.22	46.55	382.65
177.30	5.61	39.56	221.91
143.58	4.20	44.99	188.73
103.03	2.31	71.65	165.37
132.40	2.61	57.15	149.22
208.38	9.49	38.97	369.75
164.42	8.00	36.48	291.92
204.15	7.63	38.42	293.17
167.79	9.60	47.30	454.22
142.48	7.90	51.81	409.17
89.45	2.21	78.40	173.37
106.83	4.64	49.24	228.29
166.78	4.26	34.86	148.42
136.78	9.56	58.70	561.35
227.94	8.56	30.77	263.43
189.44	5.49	37.02	203.34
244.08	10.93	38.56	421.34
160.68	4.07	49.39	200.87
142.84	6.17	62.67	386.64
238.49	8.05	32.50	261.51
106.80	3.33	53.57	178.48
149.91	13.11	48.63	637.58
185.28	8.57	33.37	285.94
164.61	6.81	43.16	293.98
